# Supplementary material for: Endothelial Protein C Receptor Could Contribute to Experimental Malaria-Associated Acute Respiratory Distress Syndrome
Source: J Immunol Res. 2019 Dec 4;2019:3105817. doi: 10.1155/2019/3105817 (PMC6913256; doi:10.1155/2019/3105817)
Supplement: Supplementary Materials — Supplementary Figure 1: primary microvascular lung endothelial cell characterization. PMLECs were stained by immunofluorescence with lectin from Ulex europaeus and anti-VWF, CD31, ACE, CD62E, eNOS, and VE-cadherin antibodies. Nuclei stained with Hoechst stain (blue). PMLECs: primary culture of microvascular lung endothelial cells from DBA/2 mice (scale bar: 50 μm). Supplementary Figure 2: ARDS-developing mice show more ICAM-1 and VCAM expression in the lungs than HP-developing or noninfected mice. (a–c) DBA/2 mice infected with Plasmodium berghei ANKA and lung tissues were analyzed on the 7th day postinfection. (a) Representative immunohistochemistry images for ICAM-1 and VCAM in the lungs of NI, ARDS-developing, and HP-developing mice (scale bar: 100 μm). (b) Quantification of (b) ICAM-1 and (c) VCAM areas stained by immunohistochemistry. (d–i) Primary microvascular lung endothelial cells from uninfected DBA/2 mice (PMLECs) were stimulated with Plasmodium berghei ANKA-infected red blood cells (iRBCs) or with recombinant TNF and analyzed for (d–f) ICAM-1 mRNA and (g–i) VCAM mRNA by qRT-PCR. Bars represent the average ± SD. (b–i) One-way ANOVA from two grouped experiments (∗p < 0.05; ∗∗p < 0.01; ∗∗∗p < 0.001). ARDS: acute respiratory distress syndrome; HP: hyperparasitemia; NI: noninfected mice; NS: nonstimulated cells. Supplementary Figure 3: dexamethasone reduced inflammation and VCAM expression but did not decrease ICAM-1 expression in P. berghei-infected mice. Plasmodium berghei ANKA-infected DBA/2 mice were treated with dexamethasone and compared to infected-untreated mice. Serum and lung tissues were analyzed on the 7th day postinfection. Serum levels of (a) IL-6 and (b) IL-33 analyzed by ELISA. mRNA expression from lung tissues was evaluated for (c) VCAM and (d) ICAM-1 by qRT-PCR. Bars represent the average ± SD (∗∗p < 0.01; ∗∗∗p < 0.001). (a–b) Unpaired t-test representative of two independent experiments; (c–d) Mann-Whitney's test from two grouped experiment [file 3105817.f1.pdf]

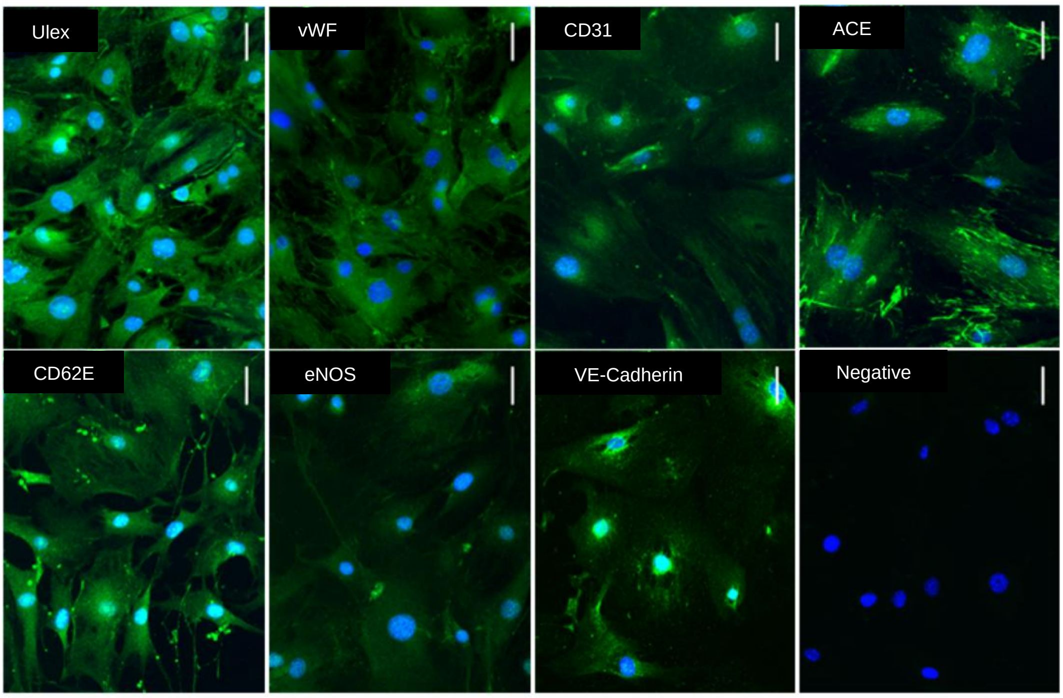

Supplementary Figure 1: Primary microvascular lung endothelial cell characterization. PMLECs were stained by immunofluorescence with lectin from *Ulex europaeus* and anti-vWF, CD31, ACE, CD62E, eNOS and VE-cadherin antibodies. Nuclei stained with Hoechst stain (blue). PMLECs: primary culture of microvascular lung endothelial cells from DBA/2 mice. (scale bar: 50 µm).

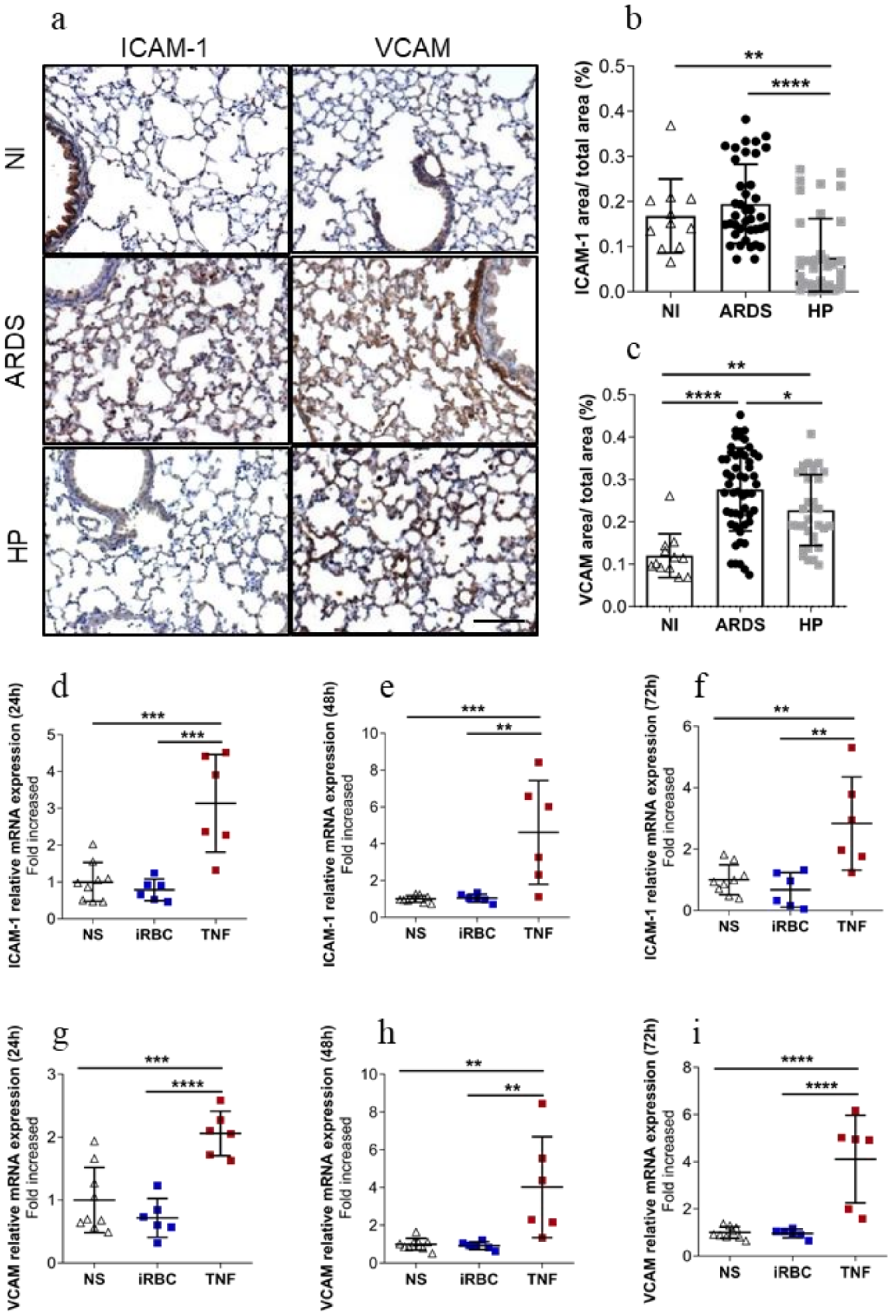

Supplementary Figure 2: ARDS-developing mice show more ICAM-1 and VCAM expression in the lungs than HP-developing or noninfected mice. (a-c) DBA/2 mice infected with *Plasmodium berghei* ANKA and lung tissues were analyzed on the 7th day post-infection. (a) Representative immunohistochemistry images for ICAM-1 and VCAM in the lungs of NI, ARDS-developing and HP-developing mice (scale bar: 100 µm). (b) Quantification of (b) ICAM-1 and (c) VCAM areas stained by immunohistochemistry. (d-i) Primary microvascular lung endothelial cells from uninfected DBA/2 mice (PMLECs) were stimulated with *Plasmodium berghei* ANKA-infected red blood cells (iRBCs) or with recombinant TNF and analyzed for (d-f)

ICAM-1 mRNA and (g-i) VCAM mRNA by qRT-PCR. Bars represent the average ± SD. (b-i) One-way ANOVA from two grouped experiments. (*$p<0.05$; ** $p<0.01$; *** $p<0.001$). ARDS: acute respiratory distress syndrome; HP: hyperparasitemia; NI: non-infected mice; NS: non-stimulated cells.

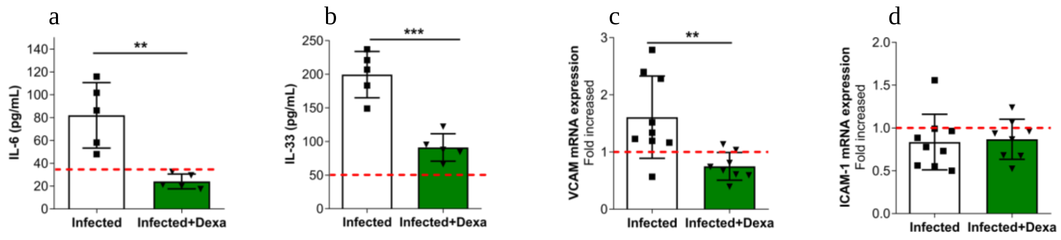

Supplementary Figure 3: Dexamethasone reduced inflammation and VCAM expression but did not decrease ICAM-1 expression in *P. berghei*-infected mice. *Plasmodium berghei* ANKA-infected DBA/2 mice were treated with dexamethasone and compared to infected-untreated mice. Serum and lung tissues were analyzed on the 7th day post-infection. Serum levels of (a) IL-6 and (b) IL-33 analyzed by ELISA. mRNA expression from lung tissues was evaluated for (c) VCAM and (d) ICAM-1 by qRT-PCR. Bars represent the average ± SD (** $p<0.01$; *** $p<0.001$). (a-b) Unpaired t test representative of two independent experiments; (c-d) Mann-Whitney test from two grouped experiments. Infected+Dexa: *P. berghei* ANKA-infected mice and treated with dexamethasone. Red dashed lines: noninfected mice.
